# CD169-Mediated Trafficking of HIV to Plasma Membrane Invaginations in Dendritic Cells Attenuates Efficacy of Anti-gp120 Broadly Neutralizing Antibodies

**DOI:** 10.1371/journal.ppat.1004751

**Published:** 2015-03-11

**Authors:** Hisashi Akiyama, Nora-Guadalupe Pina Ramirez, Manasa V. Gudheti, Suryaram Gummuluru

**Affiliations:** 1 Department of Microbiology, Boston University School of Medicine, Boston, Massachusetts, United States of America; 2 Bruker Nano Surfaces, Salt Lake City, Utah, United States of America; Vanderbilt University School of Medicine, UNITED STATES

## Abstract

Myeloid dendritic cells (DCs) can capture HIV-1 via the receptor CD169/Siglec-1 that binds to the ganglioside, GM3, in the virus particle membrane. In turn, HIV-1 particles captured by CD169, an I-type lectin, whose expression on DCs is enhanced upon maturation with LPS, are protected from degradation in CD169^+^ virus-containing compartments (VCCs) and disseminated to CD4^+^ T cells, a mechanism of DC-mediated HIV-1 trans-infection. In this study, we describe the mechanism of VCC formation and its role in immune evasion mechanisms of HIV-1. We find HIV-1-induced formation of VCCs is restricted to myeloid cells, and that the cytoplasmic tail of CD169 is dispensable for HIV-1 trafficking and retention within VCCs and subsequent trans-infection to CD4^+^ T cells. Interestingly, introduction of a di-aromatic endocytic motif in the cytoplasmic tail of CD169 that results in endocytosis of HIV-1 particles, suppressed CD169-mediated HIV-1 trans-infection. Furthermore, super-resolution microscopy revealed close association of CD169 and HIV-1 particles in surface-accessible but deep plasma membrane invaginations. Intriguingly, HIV-1 particles in deep VCCs were inefficiently accessed by anti-gp120 broadly neutralizing antibodies, VRC01 and NIH45-46 G54W, and thus were less susceptible to neutralization. Our study suggests that HIV-1 capture by CD169 can provide virus evasion from both innate (phagocytosis) and adaptive immune responses.

## Author Summary

Dendritic cells (DCs) are professional antigen presenting cells, and their sentinel roles are important to elicit a potent antiviral immunity. However, HIV-1 has exploited DCs to spread infection by several mechanisms. One such mechanism is the DC-mediated trans-infection pathway, whereby DCs transmit captured virus to CD4^+^ T cells. We have recently identified the type I interferon (IFN-I) inducible protein, CD169, as a receptor on DCs which mediates HIV-1 capture and trans-infection. We have also demonstrated extensive co-localization of HIV-1 with CD169 within peripheral non-lysosomal compartments in DCs, although the mechanism and biological importance of the compartment formation remain unclear. Here in this study, we report that a myeloid cell specific co-factor interacts with CD169 following virus capture leading to compartment formation. This co-factor is induced in DCs by an IFN-I-inducing TLR ligand LPS, but not by IFN-I itself. Though the CD169^+^ HIV-1 containing compartments are surface-accessible, these compartments have considerable depth and are connected to the surface, such that captured virus particles localized within these unique structures are protected from detection by anti-gp120 broadly neutralizing antibodies. Our study suggests that CD169–HIV-1 interaction provides an evasion mechanism from degradation by phagocytosis and neutralization by anti-viral humoral responses.

## Introduction

Myeloid dendritic cells (DCs) are professional antigen presenting cells that play sentinel roles in sensing pathogens and priming adaptive immunity [1]. HIV has, however, exploited DCs to spread to CD4+ T cells and thus DCs have been suggested to play a role in systemic HIV dissemination from peripheral mucosa to secondary lymphoid tissues [2,3]. While DCs are infected with HIV and DC-derived progeny viruses can infect CD4^+^ T cells [4–7], productive infection of DCs is limiting for several reasons including low receptor/co-receptor density, presence of cell-intrinsic restriction factors and innate sensing mechanisms eliciting anti-virus immune responses such as type I interferon secretion [8–11]. In contrast, DCs can capture HIV-1 particles and transmit captured virus to CD4^+^ T cells without establishing productive infection in DCs via a tight cell-to-cell junction called virological synapse [12], a mechanism of DC-mediated HIV-1 trans-infection, that might have evolved to bypass DC-intrinsic anti-viral responses.

Recently, our group and others have identified CD169, also known as Siglec-1, as a predominant receptor for mature DC-mediated capture of HIV-1 and subsequent virus transmission to T cells [13,14]. CD169, a type I transmembrane protein, is the largest member of the sialic-acid-binding immunoglobulin-like lectin (Siglec) family, containing 17 extracellular repeats of immunoglobulin like domain including a N-terminal V-set domain that recognizes α2–3 linked sialic acid residues, a single transmembrane domain and a short cytoplasmic tail (CT) [15]. Upon HIV-1 binding to CD169 on mature DCs, HIV-1 particles accumulate in CD81 tetraspanin^+^ compartments [13,14]. These compartments are, however, only weakly or poorly stained with endosome/lysosome markers such as CD63 and Lamp1 [16,17]. Whether or not these HIV-1^+^ compartments are connected to cell surface has been matter of intense debate [reviewed in [18]]. While early studies suggested that endocytosis of HIV-1 particles was important for efficient trans-infection of T cells [19–21], recent studies, however, have called these findings into question and have suggested that surface bound HIV-1 particles present in plasma membrane invaginations were the major source of viruses contributing to efficient DC-mediated HIV-1 trans-infection of T cells [22,23]. Interestingly, the CT of human CD169 contains 44 amino acids, and there are no defined signaling motifs or phosphorylation sites that could contribute to potential virus particle trafficking and internalization upon ligand binding. Therefore, how CD169-bound HIV-1 particles are accumulated and viral infectivity preserved in these compartments remains unclear.

In this study, we have investigated the role of CD169 in trafficking of HIV-1 in mature DCs and facilitating HIV-1 trans-infection of T cells. We found that CD169-mediated HIV-1 trafficking to non-endocytic plasma membrane invaginations is cell-type specific, and that trans-infection could be achieved even in the absence of the CT. Trans-infection efficacy was correlated with the ability of CD169 to retain HIV-1 particles on the cell surface. Interestingly, a single amino acid substitution (Ala to Tyr at position 1683) in the CT of CD169 resulted in the endocytosis of CD169-bound HIV-1 and the mutant CD169 was unable to support trans-infection of T cells, suggesting surface retention by CD169 is critical for HIV-1 to gain access to the trans-infection pathway. Furthermore, using super resolution microscopy, we observed that CD169 and HIV-1 particles were closely associated in LPS-matured DCs in compartments at the cell periphery, approximately 800 nm to 1 μm in depth from the cell surface. These peripheral virus-containing plasma membrane invaginations were not observed in DCs matured by exposure to IFN-α alone, suggesting a requirement for a LPS-inducible host co-factor for formation of the CD169^+^ HIV-1 containing plasma membrane invaginations. Intriguingly, HIV-1 particles localized within plasma membrane invaginations in LPS-matured DCs were inefficiently accessed by and hence less susceptible to α-gp120 broadly neutralizing antibodies compared to cell free viruses. Our study here, therefore, demonstrates that CD169-mediated capture and trafficking of HIV-1 within DCs can not only provide virus evasion from endocytic mechanisms that can lead to virus particle degradation in lysosomal compartments but also protect HIV-1 from neutralizing antibodies via formation of virus-containing surface-exposed plasma membrane invaginations in LPS-matured DCs.

## Results

### Surface-accessible VCC formation is specific to myeloid cells

Previous studies have reported that upon virus capture by mature DCs, HIV-1 particles accumulate in compartments at the cell periphery [23,24]. Furthermore, formation of DC–T cell conjugates results in polarized release of captured virus particles towards T cells for establishment of optimal CD4^+^ T cell infection [25]. We, as well as others, have recently reported that HIV-1 particles in these compartments are strongly colocalized with CD169 [13,14]. Since CD169 was also colocalized with HIV-1 at the DC–T cell virological synapse [13,14], we wanted to determine the mechanism by which CD169 mediates trafficking of HIV-1 particles in mature DCs.

**Fig 1:**
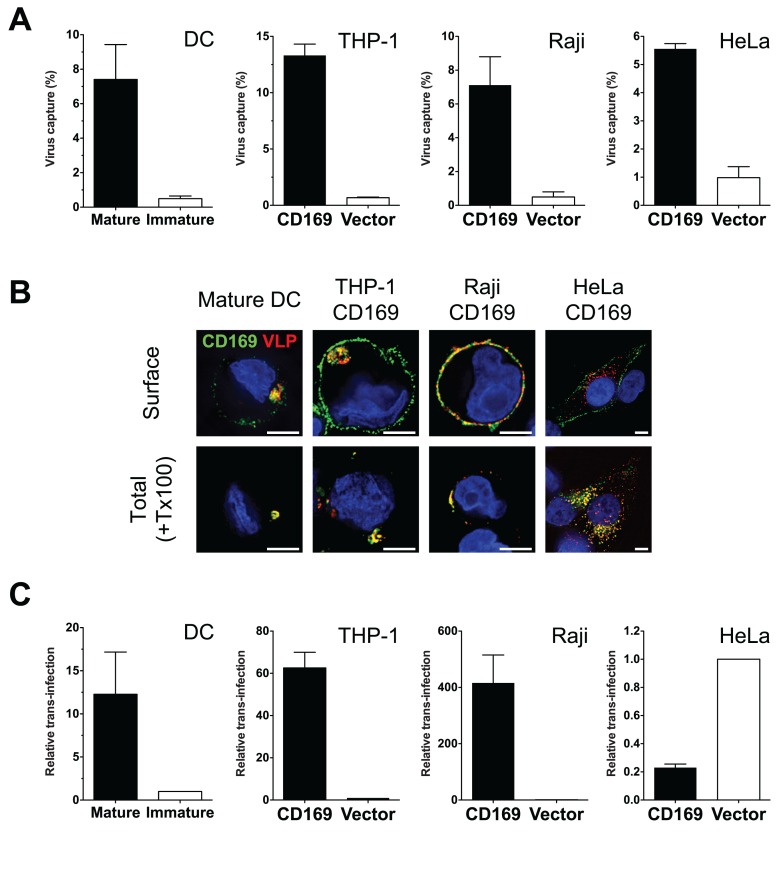
THP-1/CD169 cells recapitulate mature DC-mediated HIV-1 capture, trafficking and trans-infection of CD4^+^ T cells. (A) Cells incubated with HIV-1 particles were lysed, and cell lysates used for measuring cell-associated p24^gag^. The data shown are the mean percent of captured p24^gag^ (virus) ± SEM of independent experiments performed in triplicates (n = 3 for DC, n = 5 for THP-1, n = 3 for Raji and HeLa). (B) CD169 expressing cell lines and mature DCs were incubated with Gag-mCherry VLPs and stained for plasma membrane bound CD169 (Surface, top panel) or total CD169 (+ Tx100, bottom panel). CD169 (green), Gag-mCherry VLP (red) and nucleus (blue). Representative deconvolved images of single slices of cells are shown. Scale bars represent 5 μm. (C) Cells incubated with HIV-1 particles were co-cultured with CD4^+^ T cells to monitor HIV-1 trans-infection. Cells were lysed two days post initiation of co-culture and lysates used for measurement of luciferase activity. Values were normalized to luciferase activity observed in control cells (immature DCs or CD169^low/null^ control cell lines). The data shown are the means ± SEM of independent experiments performed in triplicates with CD4^+^ T cells from different donors (n = 3 for DC, n = 4 for THP-1, n = 3 for Raji and n = 4 for HeLa).

First, we sought to establish a cell line which could recapitulate the formation of peripheral virus-containing compartments (VCCs) that are observed upon HIV-1 capture by CD169 in mature DCs [13,14]. CD169 was stably transduced into a monocytic cell line THP-1, Raji B cell line and HeLa cells. Cell-surface CD169 expression was tested by flow cytometry and found to be comparable to or higher than that observed on mature DCs (S1 Fig A). Furthermore, induced expression of CD169 on primary cells (LPS treatment of DCs, mature DCs) or engineered expression of CD169 on cell lines (THP-1, Raji or HeLa) resulted in a dramatic enhancement in virus capture (Fig. 1A). Next, we examined if any of the cell lines were able to recapitulate the formation of CD169^+^ VCCs found in mature DC. Mature DCs, THP-1/CD169, Raji/CD169 and HeLa/CD169 cells were incubated with HIV Gag-mCherry VLPs and stained for total CD169 following membrane permeabilization with TritonX-100 (+Tx100) or without membrane permeabilization to visualize cell surface CD169 (Surface) expression (Fig. 1B). In all the cells tested, VLPs were strongly colocalized with CD169 when stained after membrane permeabilization, as reported previously [14]. In mature DCs, VLPs were often found within compartments at the cell periphery some of which were stained with CD169 without membrane permeabilization (Fig. 1B). In THP-1/CD169 cells, VLPs were strongly colocalized with CD169 in compartments similar to those found in mature DCs (Fig. 1B). Interestingly, similar to mature DCs, CD169^+^ VLP^+^ compartments in THP-1/CD169 cells were also partially accessible to surface applied anti-CD169 antibodies. While VLPs captured by Raji/CD169 cells were strongly colocalized with CD169, VLPs remained at the surface in the absence of formation of VCCs. In contrast, captured VLPs were found in intracellular CD169^+^ compartments in HeLa/CD169 cells, since anti-CD169 antibody was unable to stain VCCs without membrane permeabilization (Fig. 1B).

We next determined if differential localization of HIV-1 particles upon CD169 capture in cell lines could affect CD169-mediated trans-infection. While HIV-1 particles captured by mature DCs, THP-1/CD169 cells or Raji/CD169 cells were transmitted to CD4^+^ T cells, resulting in robust infection of T cells (Fig. 1C; trans-infection was enhanced more than 10-fold in CD169^+^ cells compared to CD169^low^ immature DCs or empty vector transduced control cell lines), HeLa/CD169 cells failed to transmit HIV-1 to T cells (Fig. 1C). These findings suggest that retention of HIV-1 particles at the cell surface upon CD169-mediated capture (Fig. 1B) is necessary for virus access to the trans-infection pathway. A corollary of these findings is that endocytosed HIV-1 particles are incompetent for CD169-mediated trans-infection.

### Cytoplasmic tail of CD169 is dispensable for HIV-1 trans-infection

**Fig 2:**
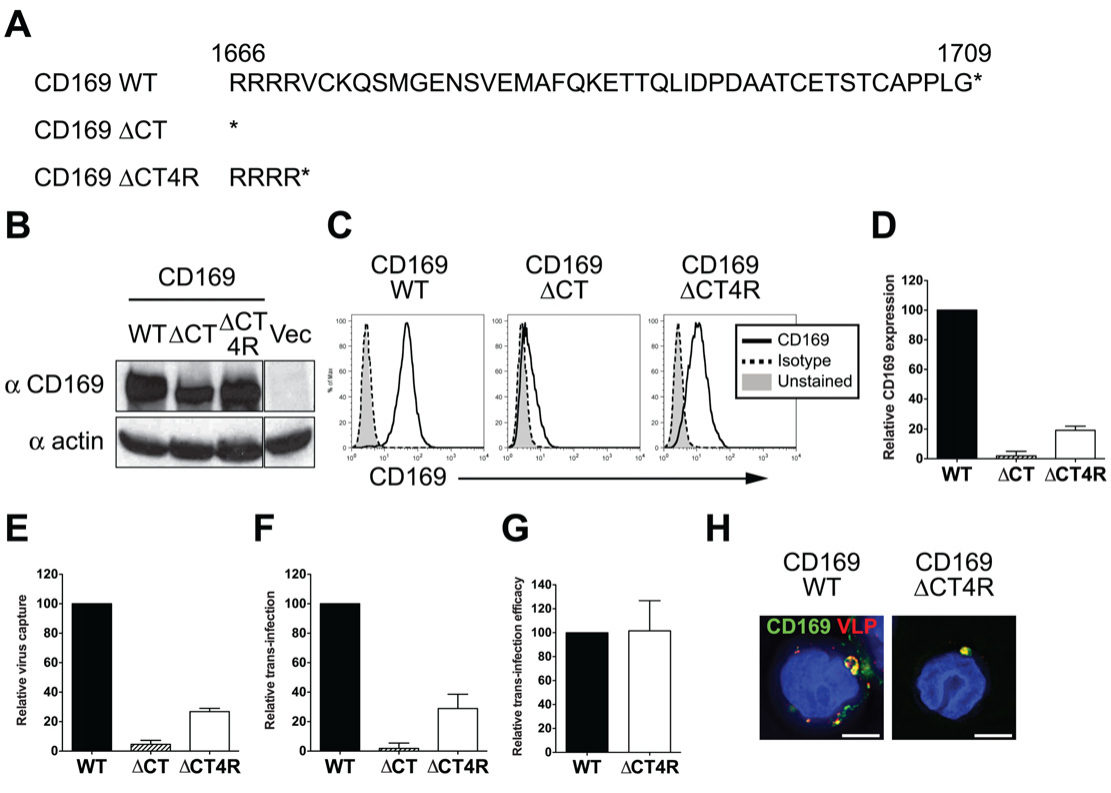
The cytoplasmic tail (CT) of CD169 is dispensable for mediating HIV-1 trans-infection. (A) Sequences of wild type and mutant CD169 CTs. The asterisks represent stop codons introduced into the ORFs of the two CT mutants. (B) Western blot analysis of THP-1 cell lysates expressing either wild type or mutant CD169. (C) Cell surface expression of CD169 on THP-1 cells was measured by flow cytometry. (D) Relative cell surface expression of CD169 CT mutants was quantified and normalized to that observed with THP-1/CD169 cells. (E) Cells were challenged with HIV-1, washed and cell-associated p24^gag^ was measured. The data shown is the virus capture by THP-1/CD169 CT mutants (ΔCT or ΔCT4R) normalized to that observed with THP-1/CD169 cells. (F) THP-1/CD169- or THP-1/CD169 CT mutant-mediated trans-infection was determined by measuring luciferase activity in THP—CD4^+^ T cell co-cultures 2 days post initiation of co-culture. The data shown is the relative virus transmission by THP-1/CD169 CT mutants (ΔCT or ΔCT4R) to that observed with THP-1/CD169 cells. (G) Efficacy of trans-infection was calculated as trans-infection (luciferase activity) per amount of virus captured (cell-associated p24^gag^) and normalized to that observed with THP-1/CD169 cells (set as 100). The data shown are the means ± SEM of three (D to F) or four (G) independent experiments. (H) THP-1/CD169 or THP-1/CD169ΔCT4R cells were incubated with Gag-mCherry VLPs (red), washed, fixed and stained for CD169 (green) and nucleus (blue). Representative deconvolved images of single slices of cells are shown. Scale bar represents 5 μm. WT: THP-1/CD169, ΔCT: THP-1/CD169ΔCT, ΔCT4R: THP-1/CD169ΔCT4R and Vec: empty vector transduced THP-1.

CD169 has been reported as a phagocytic receptor on porcine macrophages that can mediate endocytosis of PRSSV [26]. However, to date, no previously defined endocytosis signaling motifs have been described in the CT of human CD169. Since CD169 was trafficked to and colocalized with HIV-1 in surface-accessible compartments in myeloid cells (Fig. 1), we postulated that there was an unidentified trafficking motif in the CT that contributed to colocalization of CD169 and HIV-1 in VCCs. Two CD169 CT truncation mutants were constructed (Fig. 2A), one of which has a stop codon right after the transmembrane domain of CD169 (CD169ΔCT) [15]. Since previous studies have demonstrated severe reduction in cell surface expression of plasma membrane targeted proteins upon deletion of cytoplasmic tails [27,28], we constructed a second CD169 CT mutant that expressed the first four amino acids of CT (CD169ΔCT4R). These CD169 CT mutants were transduced into THP-1 cells and the ability of these stably transduced cell lines expressing CD169 mutants to capture HIV and form VCCs was compared to that observed with THP-1 cells expressing wild type CD169 (THP-1/CD169) (Fig. 1B). Deletion of the cytoplasmic tail (CD169ΔCT) resulted in decreased expression of CD169 in in THP-1 cells (Fig. 2B and S1 Fig B). Furthermore, cell surface expression of CD169ΔCT was further reduced (Fig. 2C and D) and resulted in severe attenuation of HIV-1 capture (Fig. 2E). Interestingly, inclusion of the membrane proximal 4 arginine residues in the cytoplasmic tail resulted in higher expression of CD169 in cells and partial rescue of cell surface expression of CD169 (Fig. 2C, 2D and S1 Fig B), and importantly, capture of HIV-1 particles (Fig. 2E). The efficiency of virus capture by THP-1/CD169ΔCT4R cells was much lower than that exhibited by wt THP-1/CD169 cells (Fig. 2E), in correlation with CD169 expression level on the cell surface (Fig. 2C and D). We next co-cultured CD4^+^ T cells with THP-1 cells expressing CD169 CT mutants to investigate the role of CD169 CT in mediating HIV-1 trans-infection. Interestingly, THP-1/CD169ΔCT4R but not THP-1/CD169ΔCT cells could transmit HIV-1 to CD4^+^ T cells (Fig. 2F). Furthermore, there was no significant difference in the efficiency of trans-infection (T cell infection per amount of virus captured by THP-1 cells) mediated by THP-1/CD169 and THP-1/CD169ΔCT4R cells (Fig. 2G). Finally, CD169^+^ VCCs were also observed in THP-1/CD169ΔCT4R cells (Fig. 2H), suggesting that the CD169 CT sequences downstream of the four arginine residues were dispensable for the formation of VCCs and CD169-mediated HIV-1 trans-infection.

### Enforced endocytosis of HIV-1 into endosomal compartments abrogates CD169-mediated trans-infection of T cells

**Fig 3:**
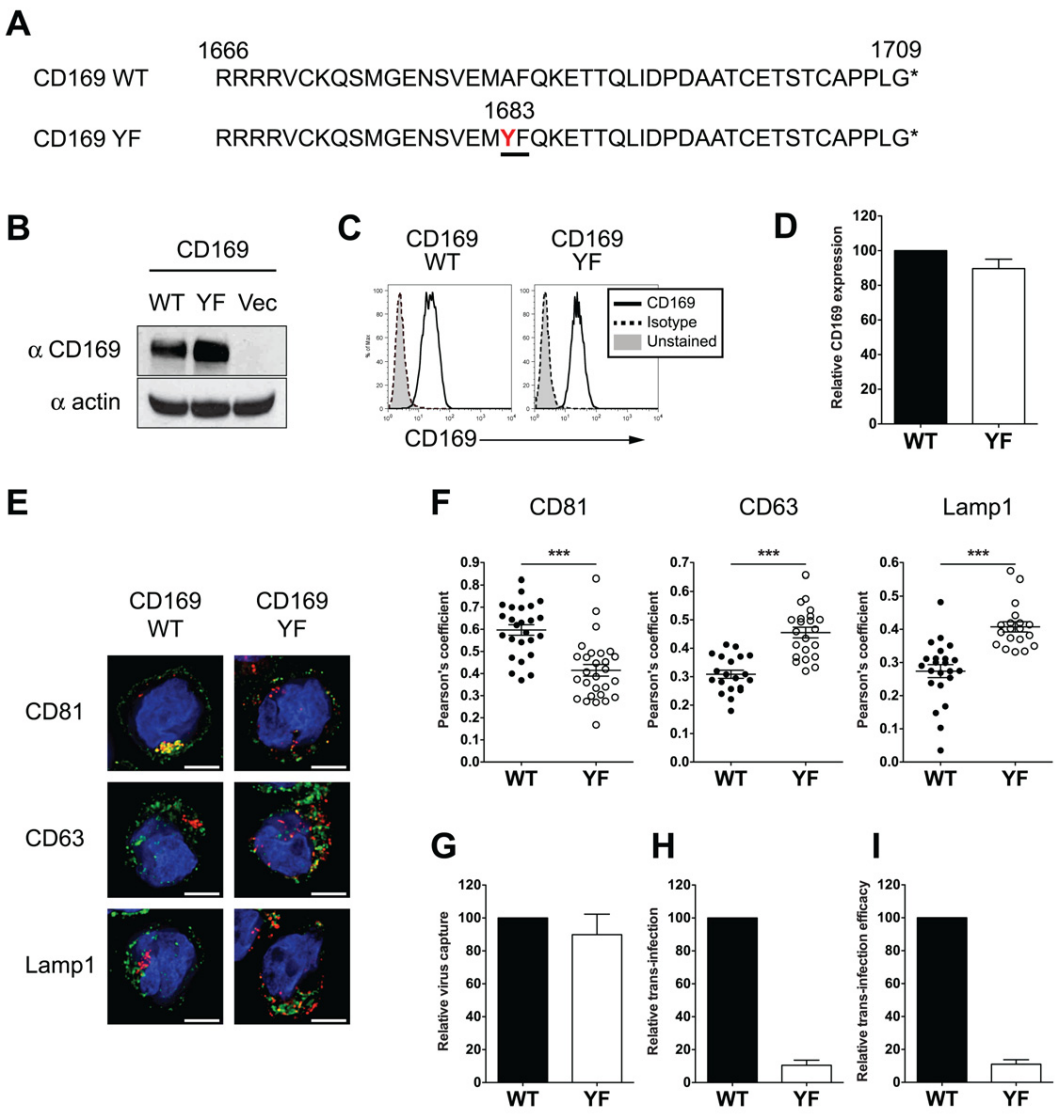
Introduction of a di-aromatic motif in CT of CD169 results in endocytosis of HIV-1 particles and attenuation of CD169-mediated trans-infection. (A) Amino acid sequences of the CTs of wild type (WT) CD169 and mutant CD169YF are shown. Alanine to tyrosine mutation at position 1683 (in red) creates a di-aromatic motif, YF (underlined). (B) Western blot analysis for CD169 expression in THP-1/CD169 and THP-1/CD169YF cell lysates. (C) Representative FACS analysis of cell surface expression of CD169 on wild type and YF mutant expressing THP-1 cells. (D) The mean fluorescence intensity of cell surface expression of CD169 on YF mutant expressing THP-1 cells was quantified and normalized to that observed with THP-1/CD169 (wt) cells (set at 100). (E) Cells were incubated with Gag-mCherry VLPs and stained for CD81, CD63 or Lamp1 and nucleus. CD81, CD63 or Lamp1 (green), Gag-mCherry VLP (red) and nucleus (blue). Representative deconvolved images of single slices of cells are shown. Scale bar represents 5 μm. (F) Co-localization between green (CD81, CD63 or Lamp1) and red (VLPs) signals is reported as mean Pearson’s coefficient ± SEM. Each dot represents a single cell. Two-tailed P values were calculated using unpaired t-test in GraphPad Prism 5. ***: P < 0.0001. (G) Cells were challenged with HIV-1, washed and cell-associated p24^gag^ was measured. Virus capture observed with THP-1/CD169YF cells was normalized to that observed with THP-1/CD169 cells (WT; set as 100). (H) Cells challenged with HIV-1/Bal-luc, were washed, co-cultured with CD4^+^ T cells and lysed at two days post initiation of co-culture for measurement of luciferase activity. The level of virus transmission observed in THP-1/CD169 (wt)—CD4^+^ T cell co-cultures was set as 100. (I) Efficacy of trans-infection was calculated as trans-infection (luciferase activity) per virus capture (cell-associated p24^gag^) and is shown relative to that observed with THP-1/CD169 cells (set as 100). The data shown are the means ± SEM of four (D) or six (G to I) independent experiments.

Whether endocytosed HIV-1 particles in DCs remain competent for trans-infection has been a matter of significant debate [6,13,16,18,21–24,29]. Since CT sequences proved dispensable for CD169 mediated trans-infection and HIV-1 particles captured by CD169 remained within surface-accessible VCCs (Fig. 2) we hypothesized that HIV-1 has exploited CD169-dependent trafficking to evade host phagocytic responses that target captured pathogens to degradative compartments. To test this hypothesis, we introduced a single point mutation in the CT of CD169 that introduces a di-aromatic motif (Ala to Tyr at position 1683) such as one known to be essential for mannose receptor-mediated phagocytosis of bacterial pathogens bearing terminal mannosylated proteins in their cell wall [30,31] (Fig. 3A). This mutant CD169, designated as CD169YF, was constitutively expressed in THP-1 cells via retroviral transduction. CD169YF expression was confirmed both by western blotting (Fig. 3B) and flow cytometry (Fig. 3C and S1 Fig B), and was expressed at similar levels at the cell surface as wild type CD169 (Fig. 3D). Interestingly, kinetics of anti-CD169 antibody internalization were enhanced in THP-1/ CD169YF compared to THP-1 cells expressing wild type CD169, suggesting the single amino acid substitution functioned as an internalization signal motif (S2 Fig A).

We next investigated the localization of HIV Gag-mCherry VLPs upon capture by THP-1/CD169YF cells. Both wt CD169 and CD169YF expressing THP-1 cells were challenged with VLPs and stained for CD81, a tetraspanin protein that colocalizes with HIV-1 in VCCs in mature DCs [16,17], or CD63 and Lamp1 (late endosomal compartment markers). In THP-1/CD169 cells, VLPs were colocalized with CD81, but not with CD63 or Lamp1 (Fig. 3E), which is consistent with previous reports on HIV-1 localization in mature DCs [16,17,23,24]. In contrast, colocalization of HIV Gag-mCherry VLPs in THP-1/CD169YF cells was reduced within CD81^+^ compartments but enhanced within CD63^+^ or Lamp1^+^ compartments (Fig. 3E). In addition, VCCs in THP-1/CD169YF were inefficiently accessed by surface-applied antibodies (S2 Fig B and C), suggesting that CD169YF internalized VLPs to late endosomes or lysosomes. These differences in intracellular localization of HIV Gag-mCherry VLPs between THP-1/CD169 and THP-1/CD169YF cells were statistically significant (Fig. 3F and S2 Fig B). While THP-1/CD169YF cells captured HIV-1 particles as efficiently as THP-1/CD169 cells (Fig. 3G), HIV-1 trans-infection of CD4^+^ T cells by THP-1/CD169YF cells was completely abrogated (Fig. 3H and I). Collectively, these results suggest that endocytosed HIV-1 particles are incompetent for accessing the CD169-dependent HIV-1 trans-infection pathway.

### HIV-1 particles in LPS-DCs are closely associated with CD169 in deep plasma membrane invaginations

**Fig 4:**
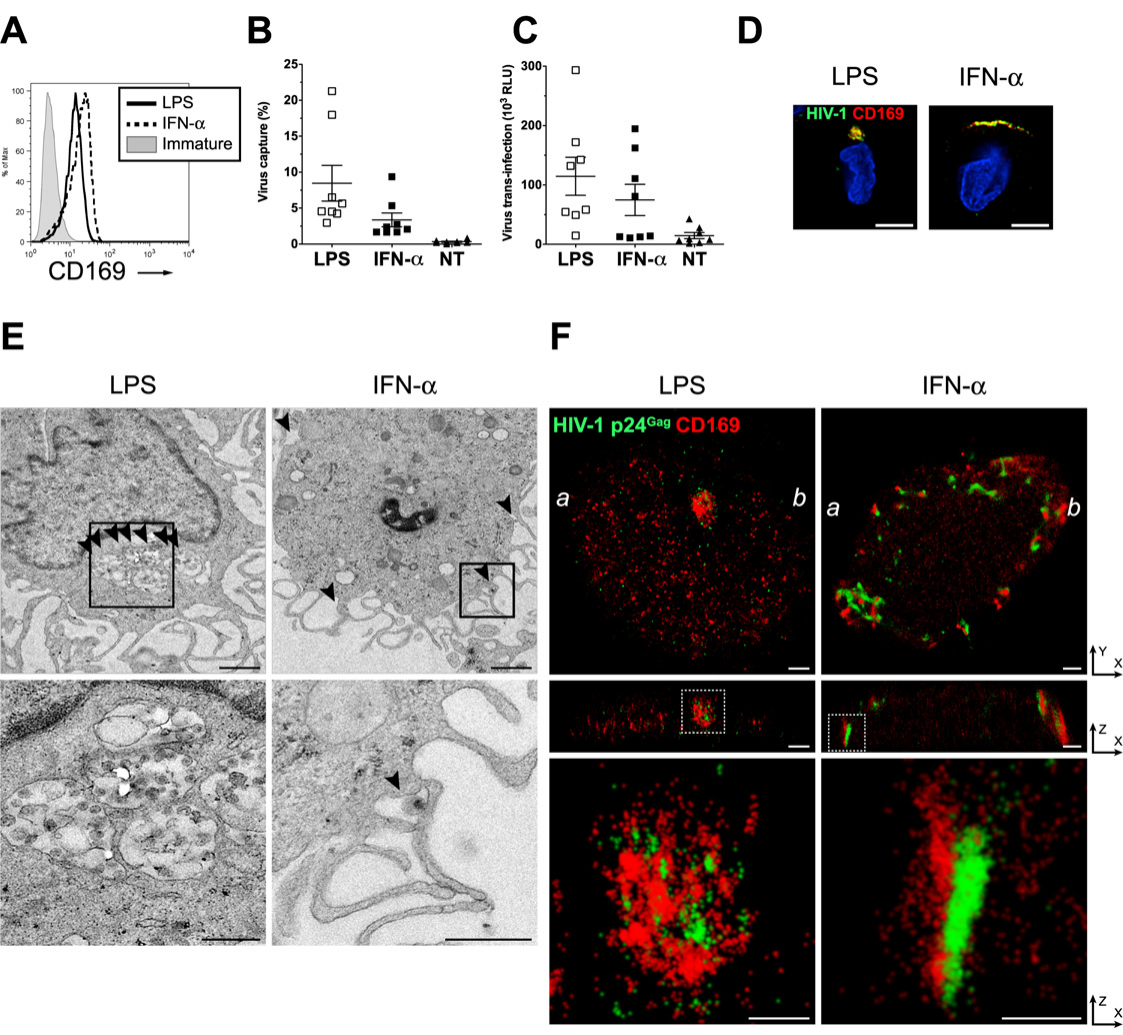
Localization of HIV-1 particles in CD169^+^ deep plasma membrane invaginations in LPS-matured DCs. (A) Representative FACS analysis for CD169 expression on LPS or IFN-α-matured DCs. (B) HIV-1 capture by immature (NT), IFN-α or LPS-matured DCs was determined by measuring cell-associated p24^gag^ in cell lysates. (C) HIV-1 transfer to CD4^+^ T cells, by immature (NT), IFN-α or LPS-matured DCs was determined by measuring luciferase activity in DC—CD4^+^ T cell co-cultures. HIV-1 capture and transfer experiments were performed in triplicates with DCs isolated from eight independent donors. The individual dot represents a single donor and the means ± SEM are depicted. (D) LPS or IFN-α-matured DCs were incubated with fluorescent HIV-1 particles (green) and stained for CD169 (red) and nucleus (blue). Representative deconvolved images of single slices of cells are shown. Scale bar represents 5 μm. (E) Representative electron micrographs of LPS or IFN-α-matured DCs incubated with HIV-1. The bottom panels are higher magnification pictures of the area depicted within the highlighted squares in the top panels. Arrows indicate virus particles. Scale bar represents 1 μm for top panels and 500 nm for bottom panels. (F) Cells were incubated with HIV-1 and stained for HIV-1 p24^gag^ (green) and CD169 (red). Cells were imaged by FPALM super resolution microscopy. The top panels represent a single LPS or IFN-α matured DC while the middle panels show cross sections along the *a*—*b* line indicated in the top panels. The bottom panels are pictures enlarged from the area depicted within the highlighted (dotted) squares in the middle panels. Scale bars represent 1 μm in the top and middle panels and 500 nm in the bottom panels. LPS: LPS-treated DCs, IFN-α: IFN-α-treated DCs, Immature: immature DCs.

We next sought to characterize the architecture in greater detail of CD169^+^ VCCs in mature DCs. CD169 expression in DCs is induced upon treatment with TLR ligands such as LPS and polyI:C [14]. As opposed to TLRs, exposure to IFN-α that results in partial maturation of DCs [32,33] can also upregulate CD169 expression [14], though putative differences in IFN-α and TLR-induced maturation phenotypes might alter virus trafficking in differentially matured DCs [34]. Therefore, DCs differentially matured with LPS or IFN-α (referred as LPS-DC or IFN-α-DC, respectively), were used for determining HIV-1 localization in phenotypically divergent CD169-expressing primary cells. While CD169 was highly upregulated on both LPS-DCs and IFN-α-DCs (Fig. 4A), IFN-α-DCs displayed a partial maturation phenotype expressing low levels of the activation antigens, CD86 and HLA-DR consistent with previously published findings [32,33]. HIV-1 capture by both LPS-DCs and IFN-α-DCs and subsequent trans-infection of CD4^+^ T cells were similarly enhanced over that observed with immature DCs (Fig. 4B and C).

We next investigated the nature of the CD169^+^ VCCs formed in IFN-α-DCs and LPS-DCs by conventional deconvolution microscopy, electron microscopy and super-resolution microscopy. While HIV-1 particles were strongly colocalized with CD169 at the cell periphery in both cell types, virus-containing pocket-like compartments were only found in LPS-DCs but not in IFN-α-DCs (Fig. 4D). Electron microscopy also revealed virus-containing pocket-like compartments in LPS-DCs as previously reported (Fig. 4E, S3 Fig A to C and [19,20]). In contrast most of HIV-1 particles were found at the surface in IFN-α-DCs (Fig. 4E and S3 Fig D to F) in valleys between dendritic extensions or present in structures presumably formed upon collapse of the dendrites in IFN- α-DCs (Fig. 4E and S3 Fig D to F). This divergent localization of HIV-1 in IFN-α- and LPS-DCs was further investigated by super resolution microscopy. We used a fluorescence photoactivation localization microscopy (FPALM) with bi-plane capture technique [35–37] which allowed us to visualize CD169^+^ VCCs in mature DCs at 20–40 nm (X-Y) and 50–80 nm (Z) resolution. In agreement with conventional deconvolution and electron microscopy (Fig. 4D and E), HIV-1 and CD169 were accumulated in pocket-like compartments in LPS-DCs, while HIV-1 was found mostly at the cellular edge in IFN-α-DCs (Fig. 4F, top panels, S4 Fig D to F and S1 Movie). Focusing at the cross section of these cells (along the line between *a* and *b* in the top panels), the depth of the compartments harboring HIV-1 particles in LPS-DCs was measured at 800 nm-1 μm (Fig. 4F, middle panels). In contrast, HIV-1 particles (p24^gag^) and CD169 clustered in a long “valley-like” structure that appeared to be on the surface of IFN-α-DCs (Fig. 4F, middle panels). In both cell types, p24^gag^ molecules (green) were closely associated with CD169 (red) (Fig. 4F, bottom panels, S4 Fig and S2 Movie and S3 Movie), implying an important role of CD169 in the formation of VCCs in DCs. All together, these results suggested that LPS and IFN-α treatment of DCs resulted in divergent CD169^+^ VCCs and that formation of CD169^+^ HIV-1 containing pocket-like structures in DCs requires a LPS-induced host factor.

### HIV-1 particles in CD169^+^ VCCs in LPS-matured DCs are surface-accessible

**Fig 5:**
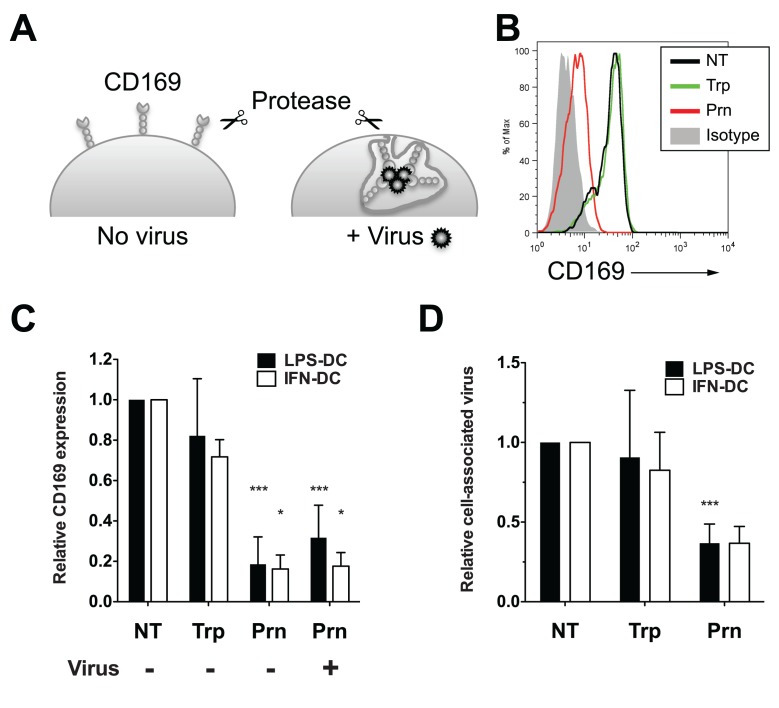
Virus particles localized within CD169^+^ VCCs in mature DCs are susceptible to pronase. (A) Experimental procedure utilized for testing the susceptibility of HIV-1 particles captured by CD169 to extracellular protease treatment is depicted. (B) Representative FACS analysis of cell surface expression of CD169 on LPS-DCs. (C) Relative cell surface expression of CD169 expression was measured by flow cytometry and normalized to that observed with untreated control (NT, set at 1). The data shown are the means ± SD of five (LPS-DCs, Trp), nine (LPS-DCs, Prn) and two (IFN-DCs, Typ and Prn) independent experiments with DCs from different donors. (D) LPS or IFN-α-matured DCs, incubated with HIV-1, were treated with pronase or trypsin. The amount of virus particles left associated with cells following protease treatments was determined by measuring cell-associated p24^gag^ and the values were normalized to that observed with untreated cells (NT). The data shown are the means ± SD of four (LPS-DCs, Trp), seven (LPS-DCs, Prn) and two (IFN-DCs, Typ and Prn) independent experiments with DCs from different donors. (C and D) Two-tailed P values were calculated using one sample t-test in GraphPad Prism 5. *: P < 0.05, ***: P < 0.0001. Trp: trypsin-treated sample, Prn: pronase-treated sample.

We next sought to determine if HIV-1 particles in CD169^+^ VCCs in LPS-DCs and IFN-α-DCs are exposed to the extracellular milieu. Uninfected or virus-exposed CD169^+^ LPS-DCs were subjected to extensive proteolytic digestion with either trypsin or pronase (Fig. 5A). Cell-surface CD169 expression and amount of HIV-1 particles that remained associated with LPS-DCs and IFN-α-DCs following protease treatment was determined by FACS and p24^gag^ ELISA, respectively (Fig. 5A). In the absence of HIV-1 binding, CD169 was mostly present at the LPS-DC and IFN-α-DC surface, and remained sensitive to cleavage by pronase but not trypsin (Fig. 5B and C, No virus), suggesting that extracellular domain of CD169 lacks trypsin-recognition sequences. Interestingly, when HIV-1 containing compartments were formed prior to pronase treatment, CD169 was still sensitive to pronase-digestion (Fig. 5C, + Virus). We next investigated if HIV-1 particles associated with CD169 in LPS-DCs and IFN-α-DCs were sensitive to protease digestion. LPS-DC- or IFN-α-DC-associated HIV-1 particles were insensitive to trypsin exposure (Fig. 5D), consistent with the findings that CD169 was trypsin resistant (Fig. 5B and C) and CD169—HIV-1 interaction is a protein (CD169)—lipid (GM3) interaction [13,14,38,39]. In contrast, consistent with the ability of pronase to effectively cleave cell-surface exposed CD169 (Fig. 5B), pronase treatment decreased LPS-DC- or IFN-α-DC-associated HIV-1 content by ~60% (Fig. 5D), suggesting VCCs were accessible to surface-applied pronase. The pronase-resistant cell-associated HIV-1 fraction might be attributed to those virus particles that either remain bound to residual CD169 (~20% of the CD169 molecules remained cell-associated even after pronase treatment; Fig. 5C), or p24^gag^ in the cytoplasm after virus fusion with mature DCs. All together, these results suggest that the majority of CD169^+^ VCCs in LPS-DCs and IFN-α-DCs remain accessible from the cell surface and thus sensitive to surface-applied pronase digestion.

### HIV-1 particles in LPS-matured DCs are less sensitive to broadly neutralizing antibodies

**Fig 6:**
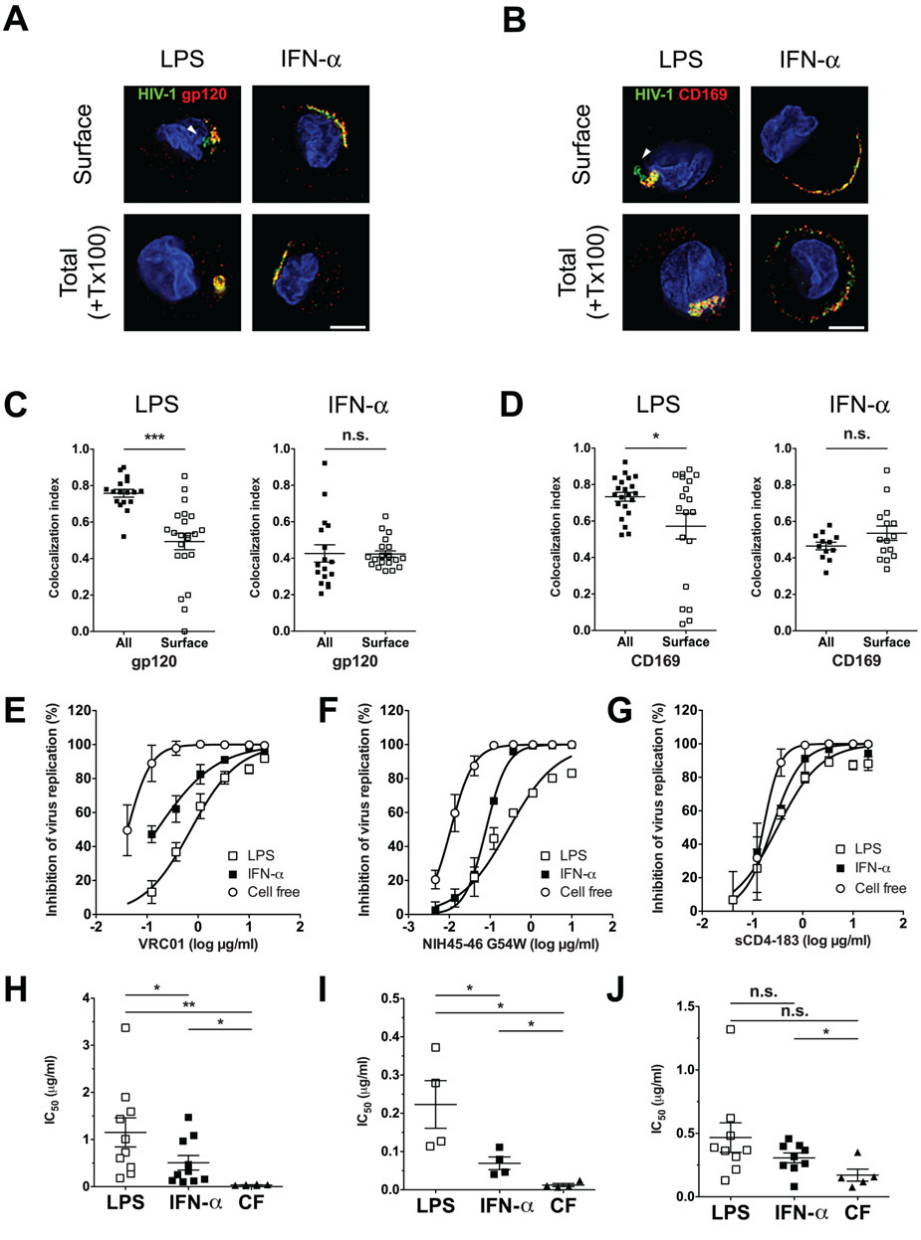
Neutralization of HIV-1 by anti-gp120 bNAbs is attenuated upon virus localization within CD169^+^ VCCs in LPS-matured DCs. (A and B) LPS or IFN-α-matured DCs incubated with fluorescent HIV-1 particles, were stained for either surface-exposed gp120 (A) or CD169 (B) on living cells (Surface, top panels) or total gp120 (A) or CD169 (B) on cells after fixation and TritonX-100 treatment (+ Tx100, bottom panels). HIV-1 particles (green), gp120 or CD169 (red) and nucleus (blue). The arrowheads indicate green HIV-1 particles in VCCs that were not stained by surface-applied antibodies. Representative deconvolved images of single slices of cells are shown. Scale bar represents 5 μm. (C and D) Co-localization between HIV-1 particles and gp120 (C) or CD169 (D) is reported as Manders’ coefficients. Each dot represents a single cell and the means ± SEM are shown. The data shown is a representative experiment using DCs isolated from two different donors. (E and F) HIV-1 exposed LPS or IFN-α-matured DCs or cell-free (CF) HIV-1 particles were incubated with increasing concentrations of VRC01 (E), NIH45–46 G54W (F) or sCD4–183 (G) prior to initiation of CD4^+^ T cell infections. The x-axis shows the concentration of input VRC01 (E), NIH45–46 G54W (F) and sCD4–183 (G) in log μg/ml, and the y-axis shows the percentage inhibition relative to infection without any antibody. The data shown are the means ± SEM of a representative experiment performed in triplicate. (H, I and J) IC50 values for VRC01 (H), NIH45–46 G54W (I) or sCD4–183 (J) are shown as mean ± SEM and each dot represents data obtained from cells derived from an independent donor. Two-tailed P values were calculated using unpaired (C and D) or paired (H, I and J) t-test in GraphPad Prism 5. * P<0.05, **: P < 0.01, ***: P < 0.0001, n.s.: not significant.

We next sought to determine if CD169-bound HIV-1 particles in CD169^+^ VCCs in LPS-DCs or IFN-α-DCs were accessible to surface-applied large molecular probes, such as anti-gp120 broadly neutralizing antibodies (bNAbs) or anti-CD169 mAbs. LPS-DCs or IFN-α-DCs were pulsed with fluorescent HIV-1 Lai-iGFP particles and stained for either HIV-1 gp120 or CD169 prior to fixation and permeabilization such that antigens accessible to surface-applied antibodies would only be visualized. As a comparison, staining for total HIV-1 gp120 or CD169 was performed in parallel after fixation and permeabilization (+ Tx100). Most of the HIV-1 particles and CD169 in IFN-α-DCs were found at the cell surface and could be visualized with surface-applied anti-gp120 (Fig. 6A) or anti-CD169 antibodies (Fig. 6B). To quantify accessibility of captured HIV-1 particles to surface-applied antibodies, the fraction of fluorescent HIV-1 particles overlapping with antibody staining was calculated using Manders' coefficients. Quantification revealed no significant differences between surface-exposed and total molecule staining for both HIV-1 gp120 and CD169 in IFN-α-DCs (Fig. 6C and D). Though some of the Lai-iGFP^+^ VCCs in LPS-DCs were stained by surface-applied anti-gp120 or anti-CD169 antibodies (Fig. 6A and B), some of the virus particles present at the “bottom” of the pocket-like structures (arrowheads, Fig. 6A and B, LPS) were inaccessible to both anti-gp120 and anti-CD169 antibody suggesting that CD169^+^ VCCs in LPS-DCs were either closed structures or inaccessible to surface-applied probes due to steric hindrance. Furthermore, we observed statistically significant differences in Manders’ coefficients amongst cells (LPS-DCs) stained by the two distinct staining techniques (Fig. 6C and D).

The differences in surface accessibility of antibodies to CD169^+^ VCCs between LPS-DCs and IFN-α-DCs prompted us to hypothesize that HIV-1 particles localized within VCCs in LPS-DCs might remain competent for mature DC-mediated trans-infection even in the presence of anti-gp120 bNAbs. To test this hypothesis, neutralization assays were performed using anti-gp120 bNAbs (VRC01 and NIH45–46 G54W) and two-domain sCD4 (sCD4–183). Either LPS-DC- or IFN-α-DC-associated HIV-1 was incubated with increasing concentrations of VRC01, NIH45–46 G54W or sCD4–183 prior to co-culture with CD4^+^ T cells. In parallel, cell free HIV-1 infection of CD4^+^ T cells was performed in the presence or absence of VRC01, NIH45–46 G54W or sCD4. While VRC01 and NIH45–46 G54W inhibited cell free CCR5-tropic HIV-1 (pseudotyped with Bal Env) infection of CD4^+^ T cells efficiently [IC_50_ (VRC01) = 0.035 ± 0.005 μg/ml (Fig. 6E and H), IC_50_ (NIH45–46 G54W) = 0.012 ± 0.004 μg/ml (Fig. 6F and I)], transfer of LPS-DC-associated HIV-1 particles was inefficiently neutralized [IC_50_ (VRC01) = 1.152 ± 0.308 μg/ml (Fig. 6E and H) and IC_50_ (NIH45–46 G54W) = 0.223 ± 0.062 μg/ml (Fig. 6F and I)]. Interestingly, transfer of HIV-1 particles from IFN-α-DCs to T cells was more susceptible to neutralization by VRC01 and NIH45–46 G54W [IC_50_ = 0.508 ± 0.155 μg/ml (Fig. 6E and H) and 0.069 ± 0.016 μg/ml (Fig. 6F and I), respectively] than that mediated by LPS-DCs, though efficiency of IFN-α-DC mediated transfer in the presence of VRC01 and NIH45–46 G54W was still greater than that observed for cell-free infection of T cells.

In contrast, sCD4–183, a small gp120-neutralizing reagent (26kD) was able to equally inhibit all three modes of infection, namely cell free, IFN-α-DC-mediated and LPS-DC-mediated HIV-1 infection of CD4^+^ T cells (Fig. 6G). The IC_50_ values for cell free, IFN-α-DC or LPS-DC-associated HIV-1 infection of CD4^+^ T cells were 0.171 ± 0.047, 0.307 ± 0.039 and 0.467 ± 0.117 μg/ml, respectively (Fig. 6J). These results suggest that HIV-1 association with CD169 in VCCs within mature DCs protects viruses from detection by anti-gp120 bNAbs and might provide virus evasion from adaptive immune responses in vivo.

## Discussion

**Fig 7:**
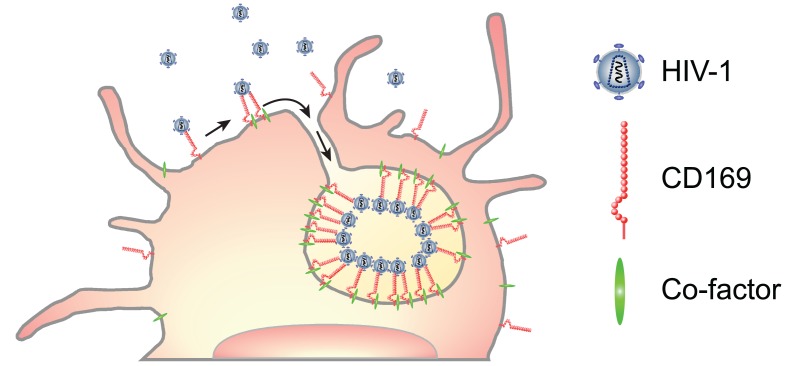
A model for CD169^+^ VCC formation in LPS-DCs. Capture of HIV-1 particles by CD169 leads to the formation of CD169^+^ VCCs in LPS-matured DCs. Lateral membrane movement of CD169-bound HIV-1 can result in accumulation of HIV-1 particles in plasma membrane microdomains in LPS-DCs. Multivalent interactions between multiple CD169 and HIV-1 particles and co-factor(s) recruitment might induce localized stress and strain to which the plasma membrane responds by forming invaginations. Arrow indicates lateral movement of CD169-bound HIV-1 particles into the VCC.

In this study, we have characterized CD169^+^ VCCs in mature DCs and found that captured HIV-1 particles in LPS-matured DCs were localized within surface-connected plasma membrane invaginations at depths of ~800nm—1μm (Fig. 4). We hypothesize that multivalent association of CD169 and HIV-1 particles or clustering of multiple CD169 molecules (induced upon virus particle binding) might enhance localized concentration of receptor-ligand complexes that are retained at the cell surface because of the inability of CD169 to mediate endocytosis. Recruitment of a LPS-induced myeloid cell-specific co-factor(s) upon virus capture to the localized membrane microdomain might place additional strain and stress on the membrane that is relieved by formation of membrane invaginations (Fig. 7), though the mechanisms that inhibit membrane closure and endosome formation remain to be identified. Interestingly, localization of HIV-1 within VCCs in LPS-matured DCs reduced the accessibility of anti-gp120 bNAb, VRC01, to virus particles and hence, reduced the neutralization efficiency of anti-gp120 bNAbs, VRC01 and NIH45–46 G54W (Fig. 6). Localization of HIV-1 and CD169 in a lattice-like structure in the VCCs might provide steric hindrance only to large molecules such as neutralizing antibodies. HIV-1 particles captured by IFN-α-DC-associated HIV-1 were also less susceptible to VRC01 and NIH45–46 G54W compared to cell free HIV-1, though localization of HIV-1 particles in IFN-α-DCs was in compartments that lacked comparable depth to that observed in LPS-DCs (Fig. 4), presumably because of the lack of recruitment of the co-factor(s) in IFN-α-DCs necessary for formation of membrane invaginations. Since HIV-1 particles were found at the bottom of dendrites and/or surrounded by dendrites (Fig. 4E) forming clusters of CD169 and HIV-1 in a "valley-like" structure (Fig. 4F), this unique localization of HIV-1 in the IFN-DCs might also hinder access of VRC01 and NIH45–46 G54W to HIV-1.

Acute infection of HIV-1 in vivo induces various pro-inflammatory cytokines including type-I interferon [40]. Such inflammatory conditions can differentiate monocytes at the site of infection into inflammatory DCs [41–43]. We have reported previously that inflammatory DCs generated in vitro are CD169^+^ and efficiently disseminate HIV-1 to T cells [14]. While triggering type I IFN responses induces the expression of number of interferon-stimulated genes, some of which are anti-viral, and restrict virus replication, induction of CD169 might offset ISG-mediated restrictions to virus replication in the peripheral mucosa. Thus, in acute phase of infection, type I IFN-induced CD169 on inflammatory DCs might support establishment of infection in mucosal CD4+ T cells. In addition to IFNs, previous studies have demonstrated increases in serum LPS levels over the course of HIV-1 infection, primarily due to the compromised integrity of gut epithelium [44]. Mucosal damage-associated-translocation of LPS might lead to systemic activation of DCs and upregulation of CD169 that not only enhances virus spread to CD4^+^ T cells, but also might provide evasion from humoral responses that develop but fail to neutralize cell-to-cell transmission in the mucosal tissues. A great deal of effort currently supports the design of viral vector-based immunoprophylactic regimens that express anti-gp120 bNAbs to induce protection in vivo [45,46]. Since DC-mediated trans-infection of CD4^+^ T cells has been suggested as an important pathway of HIV-1 dissemination in vivo [2,3], significantly increased antibody titers might be necessary in vivo to achieve neutralization of IFN-α-DC or LPS-DC-mediated HIV-1 dissemination.

CD169 is expressed exclusively on myeloid cells in vivo [15,47], and interestingly formation of CD169^+^ VCCs upon HIV-1 capture was only recapitulated in DCs and THP-1 monocytoid cell line, but not Raji B cells or HeLa cells, constitutively expressing CD169 (Fig. 1B). While HIV-1 particle associated with CD169 remained at the cell surface in Raji/CD169 cells, virus particles accumulated in intracellular, surface-inaccessible compartments in HeLa/CD169 cells. These results suggest the formation of surface-connected VCCs might require a cofactor specific to myeloid cells. Interestingly, CT sequences of CD169 proved dispensable for VCC formation, since truncation of CT downstream of the four membrane-proximal arginine residues (THP-1/CD169ΔCT4R) preserved cell surface expression of CD169 and importantly resulted in VCC formation in THP-1 cells upon HIV-1 capture (Fig. 2). Previous results from our laboratory and others have also demonstrated that HIV-1 capture by DCs is also reduced upon treatment with β-methyl-cyclodextrin, a cholesterol sequestering reagent [17,48]. Since VCC formation occurred even in the absence of CT, it is possible that lateral association and clustering of CD169 is driven by interaction of transmembrane domain of CD169 with a protein and/or lipid molecule in such cholesterol-rich plasma membrane microdomains. It is of note that expression of such cofactor(s) is regulated by LPS stimulation of DCs but not upon treatment with IFN-α alone (Fig. 4D). Future studies will be needed to identify the nature of this myeloid-cell specific co-factor by comparing TLR4-mediated (TRIF or MyD88-dependent) and IFNAR-mediated (JAK-STAT dependent) signaling pathways in myeloid cells.

CD169 is a pattern recognition receptor that captures diverse bacterial and viral pathogens by recognizing α2,3-sialylated glycoconjugates on the pathogen surface [47]. In addition to HIV-1, capture of other enveloped viruses such as murine leukemia virus, nipah and hendra hemorrhagic fever viruses by CD169 is also dependent on binding α2,3-sialylated GSLs incorporated in the virus particle membranes [14,49]. Though some studies have implicated CD169 as an endocytic receptor that mediates internalization of pathogens into early endosomes [26], CD169, unlike other members of the Siglec protein family, CD169 has no defined endocytic motifs in its CT [15,47]. Furthermore, studies described in this report suggest that HIV-1 particles captured by CD169 are not targeted for endocytosis but rather retained on the myeloid cell-surface in plasma membrane invaginations. Interestingly, exogenous introduction of a di-aromatic endocytic motif in the CT of CD169 resulted in HIV-1 internalization and dramatic attenuation of CD169-mediated HIV-1 trans-infection (Fig. 3). Collectively, these results strongly suggest a requirement for HIV-1 retention at the cell surface for accessing the mature DC/CD169-mediated trans-infection pathway.

We hypothesize that this unique trafficking pattern is beneficial to HIV-1 since it provides virus particles evasion from endocytic pathways in DCs that can result in degradation of virions and/or antigen presentation to T cells to elicit robust adaptive immune responses [50]. It is interesting to speculate that HIV-1 might have evolved to assemble and exit from GM3-enriched plasma membrane microdomains [49] such that GM3-dependent interactions of HIV-1 with CD169 provide virus sanctuary from both myeloid cell-intrinsic phagocytic mechanisms of virus degradation and antibody-dependent detection and neutralization of virus infectivity. Furthermore, as DCs have been proposed to be the first cells to encounter HIV-1 particles in the genital mucosa [2], topical administration of such reagents might prevent sexual transmission of HIV-1 Therefore, development of agents that target HIV-1–CD169 interaction might be an attractive potential anti-viral therapeutic to curtail the HIV-1 pandemic.

## Materials and Methods

### Ethics statement

This research has been determined to be exempt by the Institutional Review Board of the Boston University Medical Center since it does not meet the definition of human subjects research, since all human samples were collected in an anonymous fashion and no identifiable private information was collected.

### CD169 CT mutant construction and expression

Human CD169 was cloned into a retroviral expression vector, LNCX (LNC-CD169) and has been described previously [14]. Truncations in cytoplasmic tail of human CD169 were introduced by PCR using the following primer sets: for CD169/ΔCT, CD169–4188-sense (ATCAGGGACAGGCCATGTCC) and CD169-ΔCT-antisense (TTTTTATCGATCACCAGGTGTAGCAGGCCC CCAGG); for CD169/ΔCT4R, CD169–4188-sense and CD169-ΔCT4R antisense (TTTTTATCGATTAACGCCTCCTTCTCCAGGTGTAGCAGGC). Point mutation in the cytoplasmic tail of CD169 (A1683Y) was introduced by PCR-based site-directed mutagenesis (QuikChange; Agilent Technologies) using the following primers: CD169-YF-sense (CGAGAATTCGGTGGAGATGTATTTTCAGAAAGAGACCACGC) and CD169-YF-antisense (GCGTGGTCTCTTTCTGAAAATACATCTCCACCGAATTCTCG). A SbfI-ClaI fragment containing truncations or mutations in the CT of CD169 was replaced into the corresponding portion of LNC-CD169. All clones were verified by sequencing. Stable expression of CD169 CT mutants in THP-1 monocytic cell line, HeLa cell line and Raji B cell line was accomplished by transduction with VSV-G pseudotyped LNC-CD169 mutant retroviral vectors followed by G418 selection as previously described [14]. CD169 positive cells were further purified either by MACS (Miltenyi Biotec) or FACS (BD AriaIII). Protein expression was confirmed by western blot analysis and flow cytometry (BD Calibur) as described below.

### Plasmids

The expression plasmid, pGag-EGFP, that expresses a HIV-1 Gag-eGFP fusion protein, was obtained from the NIAID AIDS Reference and Reagent Program. HIV-1 Gag-mCherry expression plasmid that expresses a red fluorescent Gag-mCherry fusion protein has been described previously [24]. HIV-1 LaiΔenv-luc (Env deficient HIV-1 Lai containing a luciferase reporter gene in place of the nef orf), Lai/Balenv-luc (a CCR5-tropic infectious proviral construct encoding luciferase) and a CCR5-tropic infectious proviral plasmid Lai/YU-2env have been described previously [51–53]. Lai-imCherry, a proviral construct producing red fluorescent infectious virus particles, was derived from Lai-iGFP [14] by replacing the GFP-encoding fragment with that of mCherry. The CCR5-tropic HIV gp160 (Bal *env*) expression vector was generated from a CXCR4-tropic HIV gp160 (Lai *env*) expression vector [54] by replacing the entire Lai *env* gene with the corresponding region of Bal *env*.

### Cells and viruses

Human dendritic cells (DCs) were derived from CD14^+^ peripheral blood monocytes, as described previously [14]. DCs were matured with ultrapure E. coli K12 LPS (100 ng/ml; Invivogen) or IFN-α (1000 U/ml; PBL Interferon Source) for 2 days prior to use in the assays. Primary human CD4^+^ T cells were positively isolated from CD14-depleted PBMCs, using CD4-conjugated magnetic beads and LS MACS cell separation columns (Miltenyi Biotech). Positively isolated CD4^+^ T cells were activated with 2% PHA (Invitrogen) for 2 days, washed and cultured in IL-2 (50 U/ml) containing RPMI supplemented with 10% FBS. HEK293T (human kidney epithelial cell line), Raji (human B cell line, obtained from the NIH AIDS Research and Reference Reagent Program), THP-1 (human monocytic cell line, clone ATCC, obtained from the NIH AIDS Research and Reference Reagent Program), and HeLa cells have been described previously [39]. Replication competent viruses, Lai/Balenv-luc, Lai/YU-2env and Lai-imCherry, were derived from HEK293T cells via calcium phosphate transfection as described previously [54]. Fluorescent HIV Gag derived virus-like particles (VLPs) were generated via transient transfections of HEK293T cells with HIV Gag-eGFP or HIV Gag-mCherry expression plasmids. HIV-1 vectors pseudotyped with *Bal* Env were generated from HEK293T cells via co-transfection of HIV-1 LaiΔenv-luc with HIV-1 *Bal* Env expression plasmid. Viruses or VLP-containing cell supernatants were harvested 2 days post-transfection, cleared of cell debris by centrifugation (300 x g, 5 min), passed through 0.45 μm filters, and stored at—80°C until further use. For some experiments, viruses in the supernatants were concentrated by ultracentrifugation on a 20% sucrose cushion [24,000 rpm and 4°C for 2 hr with a SW32Ti rotor (Beckman Coulter)]. The virus pellets were resuspended in PBS, aliquoted and stored at -80°C. The capsid content of infectious HIV-1 particles or VLPs was determined by a p24^gag^ ELISA [54]. VSV-G pseudotyped LNC-CD169 mutant retroviral vectors were prepared as described elsewhere [14].

### Virus capture and transfer assay

Mature DCs (1x10^5^; see above), THP-1 cells (1x10^5^), Raji cells (1x10^5^) or HeLa cells (5x10^4^) were incubated with virus (10–20 ng p24^gag^) for 2 hr at 37°C in complete RPMI media, washed 4 times with PBS and analyzed for capture using either p24^gag^ ELISA. Virus capture was quantified by measuring p24^gag^ associated with lysed cells using an in-house p24^gag^ ELISA described previously [54]. For transfer of Lai/Balenv-luc infectious viruses, 1x10^5^ of mature DCs, THP-1 cells, Raji cells or 5x10^4^ HeLa cells were incubated with virus (10–20 ng p24^gag^) for 2 hr at 37°C in complete RPMI media, washed 4x with PBS and co-cultured with autologous or heterologous CD4^+^ T cells at a 1:1 or 1:2 cell ratio in complete RPMI media with IL-2. The cells were lysed at 48 hours post infection and luciferase activity in the cell lysates was measured using Bright-Glo (Promega). All assays were performed with cells derived from a minimum of three independent donors and each experiment was performed in triplicate.

### Protein analysis

To assess expression of CD169 CT mutants in THP-1 cells, cell lysates were resolved with SDS-PAGE and transferred onto PVDF membranes. Membranes were probed with mouse anti-CD169 antibody (7D2, Novus Biologicals) or rabbit anti-actin antibody (SIGMA). To measure CD169 expression on the cell surface, cells were stained with Alexa488-conjugated mouse anti-CD169 (AbD Serotec) and analyzed with a FACS Calibur (BD), as detailed in supporting methods (see S1 Text).

### Protease treatment

To determine the extent of cell-surface exposure of CD169 bound HIV-1 particles on mature DC surface, cells incubated in the presence or absence of 10 ng (p24^gag^) of HIV-1 for 2 hours were washed extensively with cold-PBS and chilled at 4°C for 30 min, prior to incubation with pronase (4 mg/ml, in Ca^2+^ containing PBS, Roche) for 30 min at 4°C. Alternatively, virus-exposed cells were incubated with 0.25% trypsin (Invitrogen) for 5 min at 37°C. After the treatment, cells were washed extensively with cold-PBS. The amount of cell-associated HIV-1 particles was measured by p24 ELISA (described above) and CD169 expression was measured by FACS as described above. The values were normalized to those of untreated samples.

### Neutralization of DC-associated virus

To investigate sensitivity of DC-associated HIV-1 to gp120-targeting neutralizing reagents, VRC01, NIH45–46 G54W and sCD4–183 (obtained from the NIH AIDS Reagent Program), 5x10^4^ mature DCs were incubated with HIV-1 Bal Env pseudotyped LaiΔenv-luc particles (10 ng p24^gag^) for 2 hours at 37°C, washed 4 times with PBS and chilled at 4°C for 15 min. Serially diluted VRC01, NIH45–46 G54W or sCD4–183 starting at 10 or 20 μg/ml in final was added to HIV-1 pulsed DCs or cell free virus (50 ng of p24^gag^) and incubated for 1 hour at 4°C. Cells were washed twice with cold-PBS and autologous or heterologous CD4^+^ T cells were added at 1:2 ratio to monitor trans-infection of CD4^+^ T cells as described above. Cell free HIV-1 was added directly to CD4^+^ T cells. These experiments were performed in triplicates with DCs from at least nine independent donors. Cell free infections of CD4^+^ T cells were performed with cells derived from at least five independent donors in triplicates. Nonlinear regression was used to estimate a fitted curve and IC_50_ values were calculated in GraphPad Prism 5.

### Super resolution microscopy

To investigate structure of HIV-1 containing CD169^+^ compartments in DCs, super resolution FPALM (fluorescence photoactivated localization microscopy) was used. LPS- or IFN-α-stimulated DCs (1x10^6^ cells) were incubated with 2.5 μg p24^gag^ Lai/YU-2env for 2 hours at 37°C and washed extensively to remove unbound viruses. Cells were fixed with 4% PFA, permeabilized, blocked with normal donkey serum and stained for HIV-1 with mouse monoclonal anti-p24 antibody (AG3.0, obtained from the NIH AIDS Reagent Program), followed by secondary donkey anti-mouse IgG-Cy3B. Cells were blocked with 20% normal mouse serum, and CD169 expression was visualized with Alexa647-conjugated anti-CD169 mAb (AbD Serotec). Cells were attached onto a grass coverslip and subjected to microscopy analysis. Images were recorded with a Vutara 200 super-resolution microscope (Bruker Nano Surfaces, Salt Lake City, UT) based on the Biplane FPALM approach [36]. Samples were imaged using a 647 nm and 488 nm excitation lasers, respectively, and 405 nm activation laser in photoswitching buffer comprising of 20 mM cysteamine, 1% betamercaptoethanol and oxygen scavengers (glucose oxidase and catalase) in 50mM Tris buffer at pH 8.0. Images were recorded using a 60x/1.2 NA Olympus water immersion objective and Photometrics Evolve 512 EMCCD camera with gain set at 50, frame rate at 50 Hz and maximal powers of 647 nm, 488 nm and 405 lasers set at 8, 10, and 0.05 kW/cm^2^ respectively. Data was analyzed by the Vutara SRX software (version 4.09). Briefly, particles were identified by their brightness from the combined images taken in both planes and two color channels simultaneously. If a particle was identified in multiple subsequent camera frames, data from these frames was combined for the specific identified particle. Identified particles were then localized in three dimensions by fitting the raw data in a customizable region of interest (typically 16x16 pixels) centered around each particle in each plane with a 3D model function which was obtained from recorded bead data sets. The four-recorded fields were aligned automatically by computing the affine transformation between each pair of planes. Sample drift was corrected by cross-correlation of the determined localized particles [55] or tracking of fiduciary markers. Fit results were stored as data lists for further analyses.

### Electron microscopy

Structure of HIV-1 containing CD169^+^ compartments in DCs was visualized by electron microscopy. 4.5 x10^6^ LPS- or IFN-α-stimulated DCs were incubated with 9 μg p24^gag^ Lai/YU-2env for 2 hours at 37°C and washed extensively to remove unbound viruses. Cells were fixed with 4% PFA and 1% glutaraldehyde in 0.1 M PHEM buffer (60 mM PIPES, 25 mM HEPES, 2 mM MgCl_2_ and 10 mM EGTA). Cells were further fixed with 2% osmium tetroxide, dehydrated in ethanol and embedded in epoxy resin as previously reported [29]. Ultra-thin sections (60–80 nm) of embedded cells were stained with 3% uranyl acetate and 1% lead citrate and subjected to imaging with a Philips CM-12 electron microscope at 100kV.

### Cellular imaging analysis

To determine if VLP containing compartments remained connected to the cell surface, HeLa/CD169 cells (seeded on coverslips in a 24-well tissue culture plate on the day before), THP-1/CD169 cells, Raji/CD169 cells or mature DCs (2x10^5^ cells) were incubated with 10 ng p24^gag^ of VLP Gag-mCherry for 2 hours at 37°C, washed extensively to remove unbound VLPs, chilled to 4°C and stained with Alexa488-conjugated mouse anti-CD169 mAb (AbD Serotec) on ice for 1 hour, prior to fixation with 4% PFA. For total CD169 staining, virus-exposed cells were fixed, permeabilized and stained with Alexa488-conjugated mouse anti-CD169 for 1 hour at RT. To determine intracellular localization of VLPs, THP-1/CD169 (2x10^5^) cells were incubated with 10 ng p24^gag^ Gag-mCherry VLPs for 2 hours at 37°C, washed and fixed with 4% PFA. Virus-containing compartments were visualized by staining with anti-human CD81 (BD), anti-human CD63 (Santa Cruz) or anti-human Lamp1 (Santa Cruz) at 10 μg/ml followed by secondary Alexa594-conjugated goat anti-mouse IgG (Invitrogen) at 10 μg/ml. To visualize HIV-1 containing compartments in DCs, LPS- or IFN-α-stimulated DCs (4x10^5^ cells) were incubated with 1 μg Lai-iGFP for 2 hours at 37°C and washed extensively to remove unbound viruses. For staining of surface-exposed HIV-1 particles or CD169, cells were chilled and stained with anti-gp120 antibody (VRC01) or anti-CD169 mAb (7D2, Novus Biologicals), respectively, at 10 μg/ml on ice for 1 hour, prior to fixation with 4% PFA. Alternatively, virus-exposed cells were fixed with 4% PFA, permeabilized with TritonX-100 and then stained with anti-gp120 antibody (VRC01) or anti-CD169 mAb (7D2, Novus Biologicals). Cells were then stained with Alexa594-conjugated goat anti-human IgG (for visualizing gp120 staining, Invitrogen) or Alexa594-conjugated goat anti-mouse IgG (for CD169 staining, Invitrogen). Nuclear staining was visualized with DAPI (Sigma) and cells were mounted on a glass slide with Fluoromount G (Southern Biotech). Images were acquired using a Olympus IX70 microscope equipped for DeltaVision deconvolution (Applied Precision). Images were deconvolved using the SoftWoRx software (Applied Precision), processed with ImageJ and pseudocolored for data presentation. For the colocalization study on THP-1 cells, images were acquired for at least 20 cells, deconvoluted, flattened for maximum intensity in order to avoid selection bias inherent in analysis of single focal plane images and analyzed for Pearson’s coefficient of correlation (R) with ImageJ. For the quantification of accessibility of antibodies to HIV-1 particles in CD169^+^ VCCs in differentially stimulated mature DCs, images were acquired on 10–15 cells, deconvolved, and flattened for maximum intensity. To specifically quantify the fraction of fluorescent HIV-1 particle overlapping with antibody signals (acquisition of red (antibody) on green (HIV-1)), Manders’ coefficients were calculated using ImageJ. Thresholds were set as the mean ± standard deviation of intensity at each channel.

## Supporting Information

S1 Fig Expression of CD169 in parental and CD169-transduced cell lines. (A) Representative FACS analysis of cell surface expression of CD169 on immature and mature DCs, parental (vector) or CD169-transduced cell lines is shown. (B) Representative FACS analysis of cell surface and total (surface and intracellular) expression of CD169 on wild type and CT mutant expressing THP-1 cells.
(EPS)

S2 Fig Introduction of a di-aromatic motif in CT of CD169 results in endocytosis of HIV-1 particles. (A) THP-1 cells expressing either wild type (WT) CD169 or CT mutant CD169YF were incubated with saturating amounts (10 μg/ml) of anti-human CD169 antibody or corresponding isotype controls for 30 minutes at 4°C. Cells were washed twice at 4°C and then an aliquot (1x10^5^ cells) was removed and placed on ice (time point 0 min). The remaining cells were then shifted to 37°C for 30 minutes to promote endocytosis. To arrest internalization, cells were transferred to 4°C, and the number of antibody-bound CD169 molecules left at the surface revealed by staining with PE-conjugated goat anti-mouse IgG antibody and analyzed by flow cytometry. The mean fluorescence intensity (MFI) of the isotype controls was subtracted at each time points and MFIs at 30 min were normalized to that observed at 0 min. The data shown is the percent of anti-CD169 antibody remaining at the cell surface 30 minutes post incubation at 37°C and is the mean ± SEM of four independent experiments. (B) Cells were incubated with Gag-mCherry VLPs and stained for plasma membrane bound CD169 (Surface, top panel) or total CD169 (+ Tx100, bottom panel). CD169 (green), Gag-mCherry VLP (red) and nucleus (blue). Representative deconvolved images of single slices of cells are shown. Scale bars represent 5 μm. (C) Co-localization between green (CD169) and red (VLPs) signals is reported as mean Pearson’s coefficient ± SEM. Each dot represents a single cell. Two-tailed P values were calculated using unpaired t-test in GraphPad Prism 5. *: P < 0.05, **: P < 0.01.
(EPS)

S3 Fig Representative electron micrographs of LPS or IFN-α-matured DCs incubated with HIV-1. High magnification images representing VCCs in LPS-matured DCs (A to C) and IFN-α-matured DCs (D to F) are shown and arrows indicate virus particles. Scale bar represents 500 nm. LPS: LPS-matured DCs, IFN-α: IFN-α-matured DCs.
(TIF)

S4 Fig Representative images of LPS or IFN-α-matured DCs incubated with HIV-1 by FPALM super resolution microscopy. (A to C) LPS-matured DCs or (D to F) IFN-α-matured DCs were incubated with HIV-1 and stained for HIV-1 p24^gag^ (green) and CD169 (red). Large images represent a single LPS or IFN-α matured DC while the insets show pictures enlarged from the area depicted within the highlighted (dotted) squares in the panels. Scale bars represent 1 μm in the large panels and 500 nm in the insets. LPS: LPS-matured DCs, IFN-α: IFN-α-matured DCs.
(TIF)

S1 Movie Colocalization of HIV-1 with CD169 on the surface of IFN-α-DCs. IFN-α-DCs were incubated with HIV-1 and stained for HIV-1 p24^gag^ (green) and CD169 (red). Z-stack images of cells were obtained via FPALM super resolution microscopy and 3D structure was reconstituted computationally. The movie represents a side view of a single cell showing HIV-1–CD169 clusters along the cell surface.
(MOV)

S2 Movie HIV-1 and CD169 are intimately associated in VCCs in LPC-DCs. LPS-DCs were incubated with HIV-1 and stained for HIV-1 p24^gag^ (green) and CD169 (red). Z-stack images of cells were obtained via FPALM super resolution microscopy and 3D structure was reconstituted computationally. The movie represents a side view of the CD169^+^ VCC shown in Fig. 4F (LPS, bottom).
(MOV)

S3 Movie HIV-1 and CD169 are clustered on the cell surface of IFN-α-DCs. IFN-α-DCs were incubated with HIV-1 and stained for HIV-1 p24^gag^ (green) and CD169 (red). Z-stack images of cells were obtained via FPALM super resolution microscopy and 3D structure was reconstituted computationally. The movie represents a side view of the CD169–HIV-1 cluster in the "valley-like" structure depicted in Fig. 4F (IFN-α, bottom).
(MOV)

S1 Text It includes information regarding the materials and methods used for determining cell surface and intracellular expression of wild type and mutant CD169 in THP-1 cells by FACS. (DOCX)
