# Correction: CD169-Mediated Trafficking of HIV to Plasma Membrane Invaginations in Dendritic Cells Attenuates Efficacy of Anti-gp120 Broadly Neutralizing Antibodies

**DOI:** 10.1371/journal.ppat.1004916

**Published:** 2015-05-07

**Authors:** The PLOS Pathogens Staff

## Notice of Republication

This article was republished on April 20, 2015 to correct the figures, which were published with low resolution. The publisher apologizes for these errors, which were introduced during the typesetting process. Please download this article again to view the correct version, with all figures now in high resolution.
