# Supplementary figures and images for: CD169-Mediated Trafficking of HIV to Plasma Membrane Invaginations in Dendritic Cells Attenuates Efficacy of Anti-gp120 Broadly Neutralizing Antibodies
Source: PLoS Pathog. 2015 Mar 11;11(3):e1004751. doi: 10.1371/journal.ppat.1004751 (PMC4356592; doi:10.1371/journal.ppat.1004751)

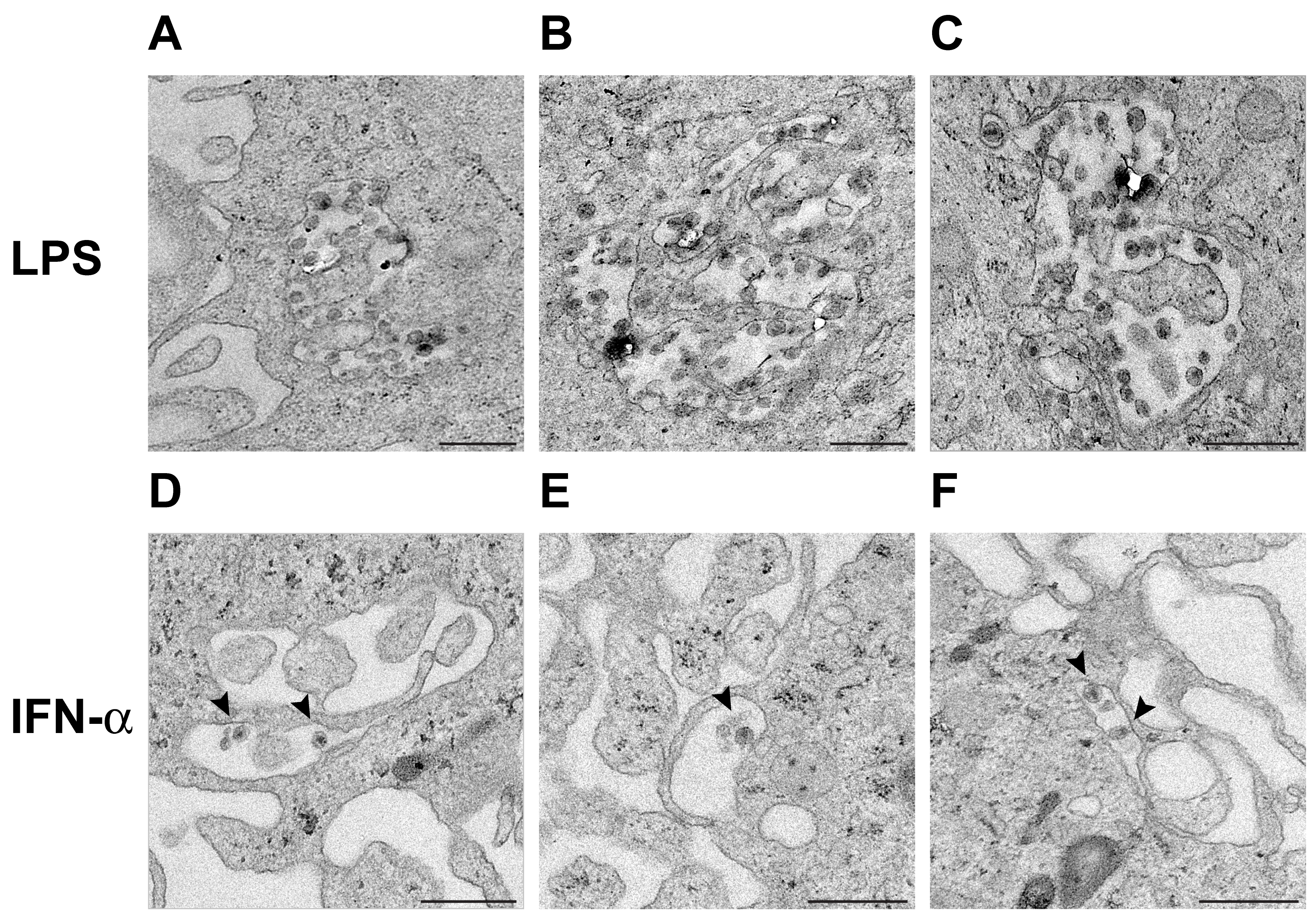

Supplement: S3 Fig — High magnification images representing VCCs in LPS-matured DCs (A to C) and IFN-α-matured DCs (D to F) are shown and arrows indicate virus particles. Scale bar represents 500 nm. LPS: LPS-matured DCs, IFN-α: IFN-α-matured DCs. (TIF) [file ppat.1004751.s003.tif]

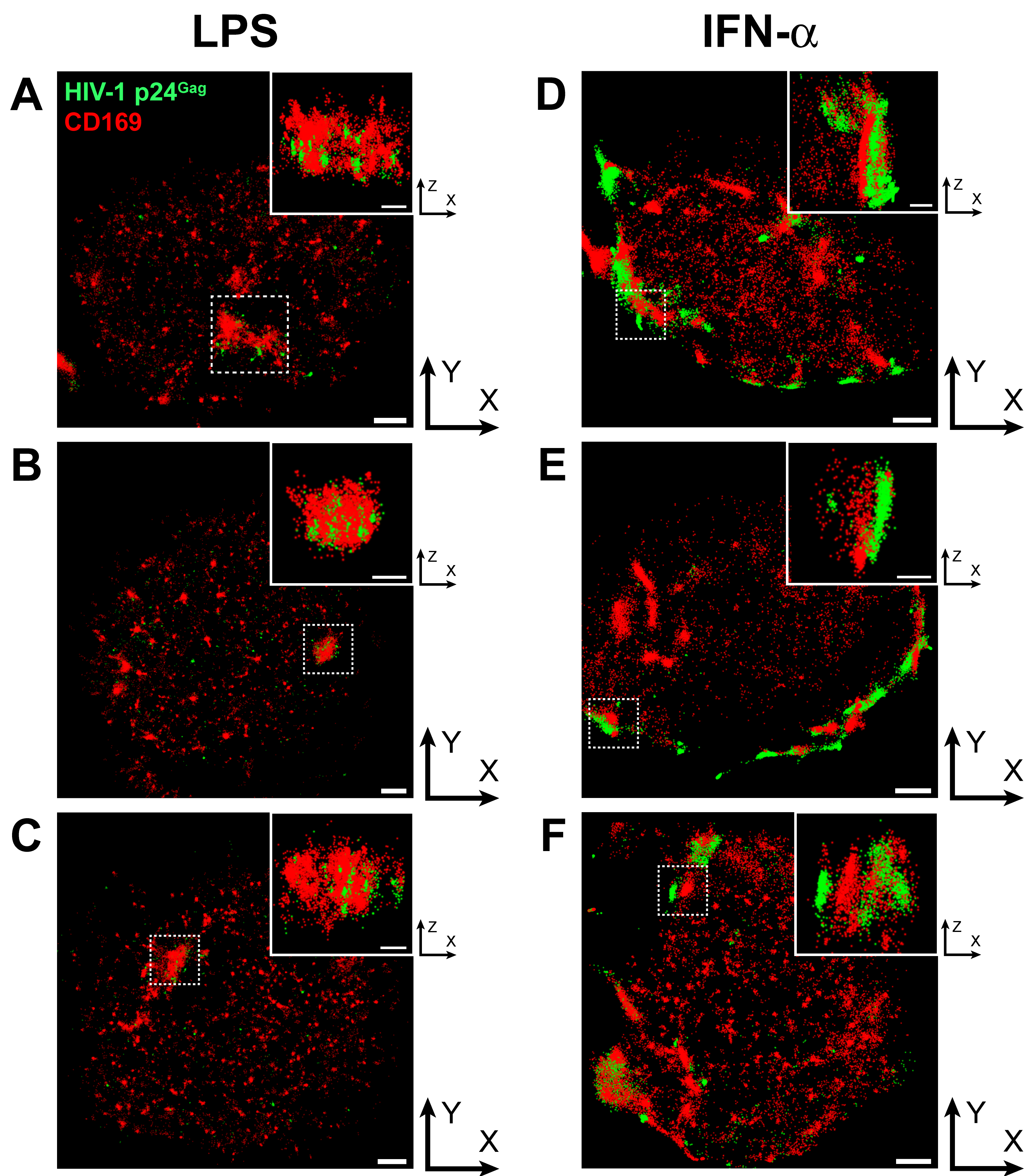

Supplement: S4 Fig — (A to C) LPS-matured DCs or (D to F) IFN-α-matured DCs were incubated with HIV-1 and stained for HIV-1 p24gag (green) and CD169 (red). Large images represent a single LPS or IFN-α matured DC while the insets show pictures enlarged from the area depicted within the highlighted (dotted) squares in the panels. Scale bars represent 1 μm in the large panels and 500 nm in the insets. LPS: LPS-matured DCs, IFN-α: IFN-α-matured DCs. (TIF) [file ppat.1004751.s004.tif]
