# Supplementary material for: CD169-Mediated Trafficking of HIV to Plasma Membrane Invaginations in Dendritic Cells Attenuates Efficacy of Anti-gp120 Broadly Neutralizing Antibodies
Source: PLoS Pathog. 2015 Mar 11;11(3):e1004751. doi: 10.1371/journal.ppat.1004751 (PMC4356592; doi:10.1371/journal.ppat.1004751)
Supplement: S1 Text — (DOCX) [file ppat.1004751.s008.docx]

**S1 Text. It includes information regarding the materials and methods used for determining cell surface and intracellular expression of wild type and mutant CD169 in THP-1 cells by FACS.**

**Supplementary materials and methods.**

**Intracellular CD169 staining**. To assess total expression of CD169 CT mutants in THP-1 cells, cells were fixed with 4% PFA, permeabilized with Perm/Wash buffer (BD), stained with Alexa488-conjugated mouse anti-CD169 (AbD Serotec) and analyzed with a FACS Calibur (BD).

**Antibody internalization assay.** Cells (5 x 10^5^) were incubated with saturating amounts (10 µg/ml) of mouse anti-human CD169 (7D2, IgG_1_, Novus Biologicals) or mouse IgG_1_ isotype control (eBioscience) for 30 minutes at 4°C in PBS containing 2% normal calf serum (NCS, GIBCO). Cells were washed twice with cold PBS/2%NCS and resuspended in RPMI complete media. An aliquot of the cells (1 x 10^5^) was removed and placed on ice as a 0 minute time point sample. The rest of the cells were then shifted to 37°C and aliquots were removed and kept on ice at 30 minutes post incubation. Surface bound mouse antibodies were revealed by staining for 30 minutes with PE-conjugated goat anti-mouse IgG antibody (Beckman Coulter) in PBS/2%NCS. Cells were washed twice, fixed in 2% PFA and analyzed with FACS Calibur (BD).
